# Persistent and Compartmentalised Disruption of Dendritic Cell Subpopulations in the Lung following Influenza A Virus Infection

**DOI:** 10.1371/journal.pone.0111520

**Published:** 2014-11-14

**Authors:** Deborah H. Strickland, Vanessa Fear, Seth Shenton, Mathew E. Wikstrom, Graeme Zosky, Alexander N. Larcombe, Patrick G. Holt, Cassandra Berry, Christophe von Garnier, Philip A. Stumbles

**Affiliations:** 1 Telethon Institute for Child Health Research and Centre for Child Health Research, University of Western Australia, Perth, W.A., Australia; 2 School of Veterinary and Life Sciences, Murdoch University, Perth, W.A., Australia; 3 School of Medicine, University of Tasmania, Hobart, Tasmania, Australia; 4 Queensland Children's Medical Research Institute, University of Queensland, Brisbane, Qld., Australia; 5 Pulmonary Medicine, Bern University Hospital and Department of Clinical Research, Berne University, Berne, Switzerland; Hannover School of Medicine, Germany

## Abstract

Immunological homeostasis in the respiratory tract is thought to require balanced interactions between networks of dendritic cell (DC) subsets in lung microenvironments in order to regulate tolerance or immunity to inhaled antigens and pathogens. Influenza A virus (IAV) poses a serious threat of long-term disruption to this balance through its potent pro-inflammatory activities. In this study, we have used a BALB/c mouse model of A/PR8/34 H1N1 Influenza Type A Virus infection to examine the effects of IAV on respiratory tissue DC subsets during the recovery phase following clearance of the virus. In adult mice, we found differences in the kinetics and activation states of DC residing in the airway mucosa (AMDC) compared to those in the parenchymal lung (PLDC) compartments. A significant depletion in the percentage of AMDC was observed at day 4 post-infection that was associated with a change in steady-state CD11b^+^ and CD11b^−^ AMDC subset frequencies and significantly elevated CD40 and CD80 expression and that returned to baseline by day 14 post-infection. In contrast, percentages and total numbers of PLDC were significantly elevated at day 14 and remained so until day 21 post-infection. Accompanying this was a change in CD11b^+^and CD11b^−^ PLDC subset frequencies and significant increase in CD40 and CD80 expression at these time points. Furthermore, mice infected with IAV at 4 weeks of age showed a significant increase in total numbers of PLDC, and increased CD40 expression on both AMDC and PLDC, when analysed as adults 35 days later. These data suggest that the rate of recovery of DC populations following IAV infection differs in the mucosal and parenchymal compartments of the lung and that DC populations can remain disrupted and activated for a prolonged period following viral clearance, into adulthood if infection occurred early in life.

## Introduction

Continuous exposure of the respiratory tract to environmental antigens poses a major challenge to the maintenance of local immunological homeostasis at this site. Inhaled foreign proteins and pathogens must be efficiently screened by the immune system for their potential “danger” to the host and either ignored in the case of harmless proteins (ignorance or tolerance), or translated into signals for induction of innate and adaptive immunity in the case of pathogens such as respiratory viruses. There is a close association between respiratory viral infections, bronchiolitis, wheezing and development of allergic asthma, particularly a subset of susceptible infants and children [1]. Human Rhinovirus (HRV), Respiratory Syncytial Virus (RSV) and Influenza A Virus (IAV) have high burdens of hospitalisation in children younger than 5 years, and particularly in those under 2 years of age [2], [3]. Airways inflammation resulting from viral infections in infancy have been linked to wheezing in pre-school years, with associations for IAV, RSV and to a lesser extent other respiratory viral infections being documented [4]-[6]. Although the development of allergic asthma involves a complex series of interactions between genes and environment, there is data associating respiratory viral infections and atopy to the development of asthma, particularly in children with atopic sensitisation by the age of 2 years [3]. While the underlying pathogenesis of virally induced allergic asthma remains unclear, experimental evidence suggests that viral infections disrupt tolerance to aeroallergens across mucosal barriers together with enhanced pro-allergic immune responses [7], [8].

Under normal circumstances, immunological homeostasis within the respiratory tract is maintained via the surveillance activities of local dendritic cell (DC) populations. These are distributed within respiratory tissues as integrated networks, playing a crucial role in sampling of inhaled antigens including viruses and allergens and in the initiation of subsequent tolerance and/or adaptive T cell-mediated immune responses in draining lymph nodes (DLN) [9]. Earlier observations from our group in a rat model were the first to demonstrate the rapid expansion of airway mucosal DC (AMDC) during acute viral (parainfluenza) infection, and the apparent persistence of this response beyond viral clearance [10]. This was subsequently confirmed in a mouse RSV model with respect to whole lung DC and similar observations have been reported in humans for nasal mucosal DC populations in children post RSV and HRV infections [11], [12]. Given the key role that these frontline DC populations in local immune surveillance for all classes of environmental antigens, their long-term disruption following viral infection has significant implications in regards to maintenance of general immunological homeostasis within the respiratory tract. With this in mind we have sought to confirm and extend these earlier observational studies, aiming to more comprehensively identify which DC subsets are susceptible to the effects of virus, and in which precise tissue microenvironments within the respiratory tree; moreover we have extended the studies to encompass immunologically immature weanling animals, similar to the age range described above for maximal susceptibility to severe virus associated airways inflammation in humans.

Early studies in rodent models demonstrated the capacity of respiratory tract DC to direct the outcome of CD4^+^ T cell responses to inhaled allergens and a number of subsequent studies have confirmed the essential requirement for DC migration to DLN for induction of CD4^+^ T cell mediated allergic airways inflammation and asthmatic syndromes [13], [14]. Furthermore, our previous work in rodents identified a subdivision of function based on anatomical location, with AMDC being functionally distinct from their counterparts in parenchymal lung tissue, most likely due to micro-environmental differences between these anatomical locations [15], [16]. Consistent with airway mucosal surfaces being the first site of exposure to inhaled allergens and viruses, AMDC show high levels of endocytic activity and rapid turn-over and drainage to airway DLN, defining their proposed role as “gatekeepers” for the initiation of adaptive immunity to inhaled allergens and pathogens [15], [17]-[19].

A number of DC subsets have been described in rodents and humans with differing capacities to influence naïve and memory CD4^+^ and CD8^+^ T cell responses [20]. In the mouse, major populations of classical (also termed “myeloid” or “conventional”) DC (cDC) and plasmacytoid DC (pDC) have been identified, as well as a number of cDC subsets with distinct phenotypic and transcriptional profiles [21]. In the mouse respiratory tract, two dominant cDC subsets based on the reciprocal expression of CD11b and the alpha (E) integrin CD103 have been described, whereby CD11b^lo^ (CD103^+^) DC express tight junction proteins, reside within the airway epithelium and increase in numbers during allergic airways inflammation, while the CD11b^hi^ (CD103^−^) subset readily produces a number of chemokines that regulate CD4^+^ T cell activity [16], [22], [23]. During IAV infection, both DC subsets have been shown to be infected and be capable of presenting viral antigens to T cells, however the CD11b^lo^ subset appears to be the predominant subset for migration to DLN and for either direct or cross-presentation of viral antigens to CD8^+^ T cells although, other subsets in tissue and DLN are likely to be involved [24]–[27]. In addition to cDC, other non-myeloid lineages such as plasmacytoid DC (pDC) in lungs, as well as resident lymphoid-origin DC in DLN, may also play important roles in initiating T cell immunity to IAV [27]–[29].

In this study we have characterised the impact of IAV infection on the frequency and activation of cDC in anatomical compartments of the respiratory tract of BALB/c mice following IAV infection. IAV infection was shown to have a differential impact on DC numbers and activation status in airway mucosal and parenchymal lung tissues, which represent two anatomically and immunologically distinct compartments of the respiratory tract. Furthermore, IAV infection of juvenile mice induced long-term alterations on the frequency and activation states of parenchymal lung DC subsets that persisted into adulthood.

## Results

### Clinical features of the BALB/c mouse model of influenza A virus (IAV) infection

**Figure 1:**
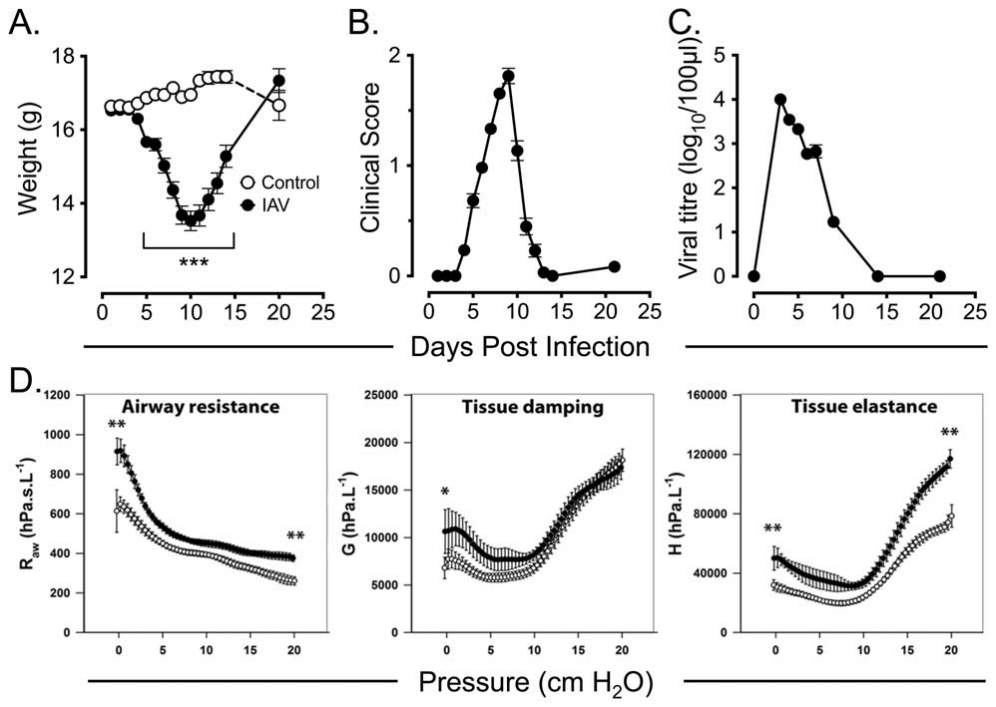
Clinical features of the adult BALB/c mouse model of A/PR8/34 H1N1 influenza A virus (IAV) infection. Eight week-old female BALB/c mice were inoculated i.n. with 1×10^2^ TCID_50_ A/PR/8/34 influenza A virus (IAV) in PBS and assessed at the indicated time points over the following 21 days for *(A)* body weight *(B)* clinical score and *(C)* lung tissue viral titres. Data are mean +/− SEM of groups of 20 to 60 mice per time point for weight measurements and clinical score, and 5 mice per time point for lung viral titres. Control mice received equivalent volumes of virus-free DMEM i.n. at day 0. *(D)* Measurement of airway resistance, tissue damping and tissue elastance at transrespiratory pressure during slow inflation manoeuvres up to 20cm H_2_O in influenza infected mice (closed circles) and controls (open circles) at day 4 post infection. Influenza infected mice had impairments in Raw, G and H. Asterisks indicate statistical significance of IAV infected as compared to control mice as described in Materials and Methods. Data are means +/− SEM of 4 mice per group. *  =  *p*<0.05; **  =  *p*<0.01; ***  =  *p*<0.001.

An adult BALB/c mouse model of IAV infection was established, using intranasal (i.n.) delivery of an optimised dose of the mouse-adapted A/PR/8/34 (H1N1) type A influenza virus (IAV). Using this protocol, mice developed peak clinical symptoms on d9 to d10 post-infection (p.i.) as evidenced by body weight loss (Fig. 1A) and clinical score (Fig. 1B), with mice recovering to pre-infection clinical score and weight by d14 and d21 p.i. respectively. Lung tissue viral titres were elevated at d3 p.i. and completely resolved by d14 p.i. (Fig. 1C). Lung mechanics were also altered at d4 p.i., with IAV infected mice showing increased airway resistance at functional residual capacity (FRC) (*p*<0.001), which was maintained throughout inflation up to 20 cmH_2_O transrespiratory pressure (*p*<0.001) (Fig. 1D, left panel). Similarly, IAV infected mice had increased tissue damping (0 cm H_2_O, *p*<0.01) (Fig. 1D, middle panel) and tissue elastance (0 cm H_2_O, *p*  =  0.002; 20 cm H_2_O, *p* <0.001) (Fig. 1D, right panel), although at high pressures there was no difference in tissue damping between influenza infected mice and controls.

**Figure 2:**
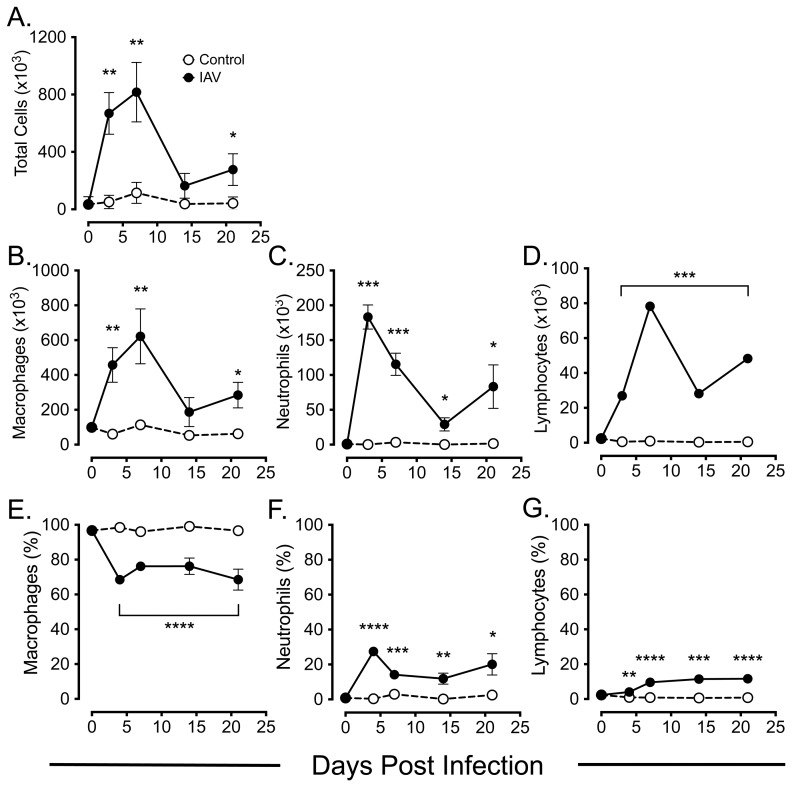
Bronchoalveolar lavage fluid (BALF) differential cell counts following IAV infection. Groups of 8 week old BALB/c mice were infected with IAV i.n. and BALF harvested on day 0 (pre-infection) and at the indicated time post-infection and assessed for total numbers of *(A)* total cells, *(B)* macrophages, *(C)* neutrophils and *(D)* lymphocytes and for percentages of cells by differential Leishman's staining for *(E)* macrophages, *(F)* neutrophils and *(G)* lymphocytes as described in Materials and Methods. Control mice received equivalent volumes of virus-free DMEM i.n. at day 0. Results are mean +/− SEM for 5 mice at each time point. *  =  *p*<0.05; **  =  *p*<0.01; ***  =  *p*<0.001; ****  =  *p*<0.0001.

Total cell counts in bronchoalveolar lavage fluids (BALF) from IAV infected mice showed a significant increase at d4 p.i. (*p* <0.01), and peak increase at d7 p.i. (*p*<0.01), when compared to control mice, declining by d14 p.i. but still remaining significantly elevated above control levels at d21 p.i. (*p*<0.05). (Fig. 2A) Differential cell counts of BALF showed significantly elevated macrophage numbers at d4 p.i. (*p*<0.01) that mirrored the kinetics of total cell counts (Fig. 2B). An early neutrophil response was observed, with a peak at d4 p.i. (*p* <0.0001) that declined but remained significantly elevated above control levels at d14 p.i. (*p* <0.05), and at d21 p.i. (*p*<0.05) (Fig. 2C). Total lymphocyte counts were also significantly elevated by d4 p.i. (*p*<0.0001) with a peak at d7 p.i. (*p*<0.0001), declining but remaining significantly elevated above control levels at d14 and d21 p.i. (*p*<0.0001). (Fig. 2D). Calculation of the percentage of each cell type in BALF showed a significant decrease in the percentage of macrophages from d4 to d21 p.i. (Fig. 2E), and significant increases in the percentages of neutrophils (Fig. 2F) and lymphocytes (Fig. 2G) over the same time period.

**Figure 3:**
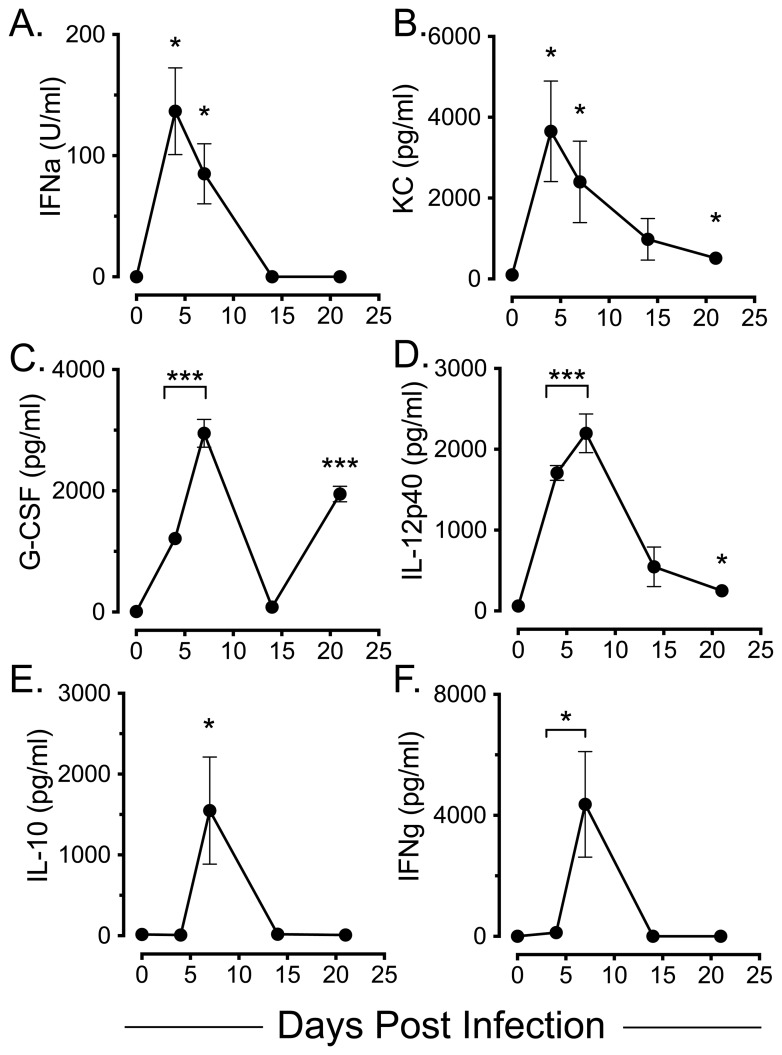
BALF cytokine analysis following IAV infection. Mice were infected with IAV i.n. and BALF fluids harvested at the indicated time points and assessed for *(A)* IFNα by bioassay as described in Materials and Methods, and by multiplex immunoassay assay for *(B)* KC, *(C)* G-CSF *(D)* IL-12 p40, *(E)* IL-10 and *(F)* IFNγ. Results shown are means +/− SEM of duplicate samples for 3 mice at each time point. Statistical significances were calculated for infected mice relative to control (d0) mice. *  =  *p*<0.05; ***  =  *p*<0.001.

Cytokine levels in BALF showed significantly elevated IFNα (Fig. 3A) and KC (Fig. 3B) levels at d4 p.i. (*p*<0.05) that declined to control levels by d14 for IFNα, whereas KC remained elevated until d21p.i. (*p*<0.05). G-CSF showed a biphasic response, being significantly elevated at d4 and d7 p.i. (*p*<0.0001), returning to baseline levels at d14 p.i. and significantly increasing again at d21 p.i. (*p*<0.0001) (Fig. 3C). IL-12 p40 (but not IL-12p70, data not shown) was significantly elevated at d4 and d7 p.i. (*p*<0.0001), declining but remaining significantly elevated at d21 p.i. (*p*<0.05) (Fig. 3D). Both IL-10 (Fig. 3E) and IFNγ (Fig. 3F) were significantly elevated (*p*<0.05) at d7 p.i., returning to control levels by d14 p.i.

### Kinetics of airway mucosal and lung parenchymal DC subsets following acute IAV infection

**Figure 4:**
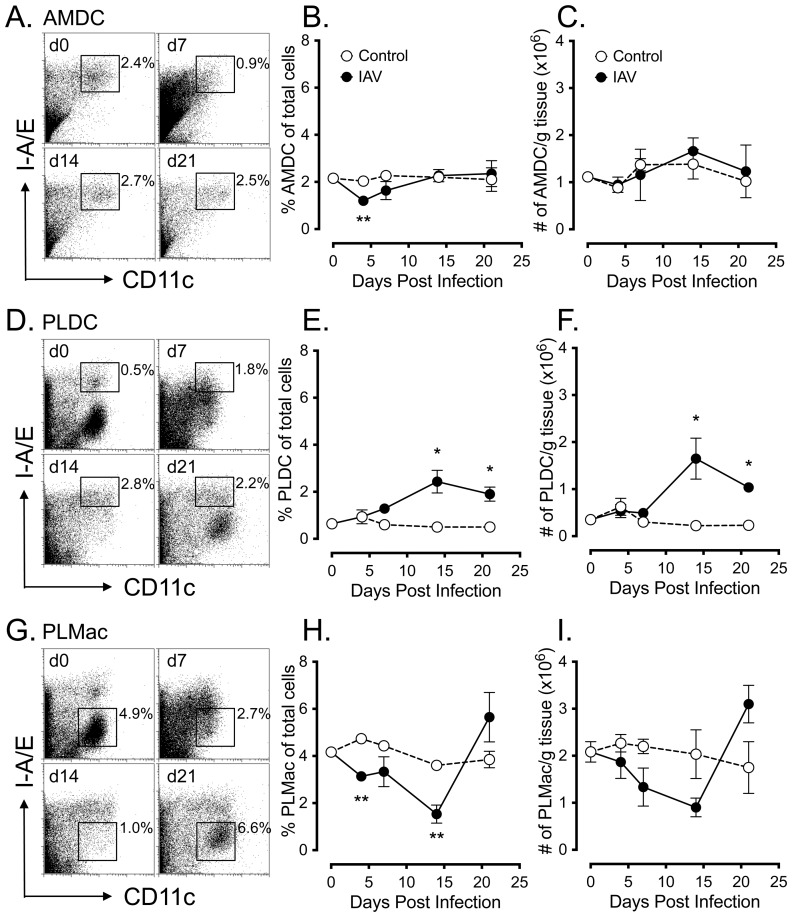
Kinetics of airway mucosal and parenchymal lung DC changes following IAV infection. *(A)* Representative FACS profiles showing gating for AMDC at the indicated time points p.i. *(B and C)* AMDC percentage frequencies *(B)* and total numbers *(C)* following IAV infection for control (open circles) and IAV infected mice (closed circles). *(D)* Representative FACS profiles showing gating for PLDC at the indicated time points p.i. *(E and F)* PLDC percentage frequencies *(E)* and total numbers *(F)* following IAV infection for control (open circles) and IAV infected mice (closed circles). *(G)* Representative FACS profiles showing gating for PLMac at the indicated time points p.i. *(H and I)* PLMac percentage frequencies *(H)* and total numbers *(I)* following IAV infection for control (open circles) and IAV infected mice (closed circles). Data are means +/− SEM for 3 independent infection experiments using pools of tissue from 3 to 4 mice for each experiment. *  =  *p*<0.05; **  =  *p*<0.01.

Our previous mouse studies have identified functionally distinct populations of DC in the airway mucosa (AMDC) and parenchymal lung (PLDC), with AMDC displaying more rapid turnover rates (<12 h) compared to PLDC (>7 days) and more rapid activation in response to aeroallergen challenge [15], [16]. Given that acute IAV infection is characterised by early infection and replication of the virus in epithelial cells of the airway mucosa, we initially examined the population dynamics of AMDC compared to their more peripheral PLDC counterparts following IAV infection. AMDC and PLDC were identified by flow cytometry using co-staining of tracheal and parenchymal lung tissue respectively for CD11c and MHC class II (I-A/E) as previously described [15], allowing gating of CD11c^+^ I-A/E^hi^ AMDC and PLDC following IAV infection (Figs. 4A and 4D). This combination of markers also allowed identification of CD11c^+^ I-A/E^low^ parenchymal lung macrophages (PLMac) [15], which were also tracked over the same time course post-IAV infection (Fig. 4G). In addition, as expression of I-A/E may possibly have modulated after IAV infection, we confirmed that these phenotypes remained stable by substituting the mouse DC marker CD205 for I-A/E (Fig. S1). A time course analysis of tracheal tissue showed a significant depletion of AMDC as a percentage of total cells (*p*<0.01) (Fig. 4A and 4B), but not total cell numbers (Fig. 4C), at day 4 p.i. with the percentages of AMDC returning to baseline levels by d7-14 p.i. In contrast, percentages (Fig. 4D and 4E) and total numbers (Fig. 4F) of PLDC in peripheral lung tissue remained unchanged from controls at d4 p.i., but were then significantly increased above control levels from d14 p.i (*p* <0.05). Over the same time-course, a decrease in percentages (Fig. 4G and 4H) and total numbers (Fig. 4I) of PLMac was observed from d4 p.i., with a significant decrease in the percentage of PLMac at d4 and d14 p.i. (*p*<0.01), returning to near-baseline levels at d21 p.i.

**Figure 5:**
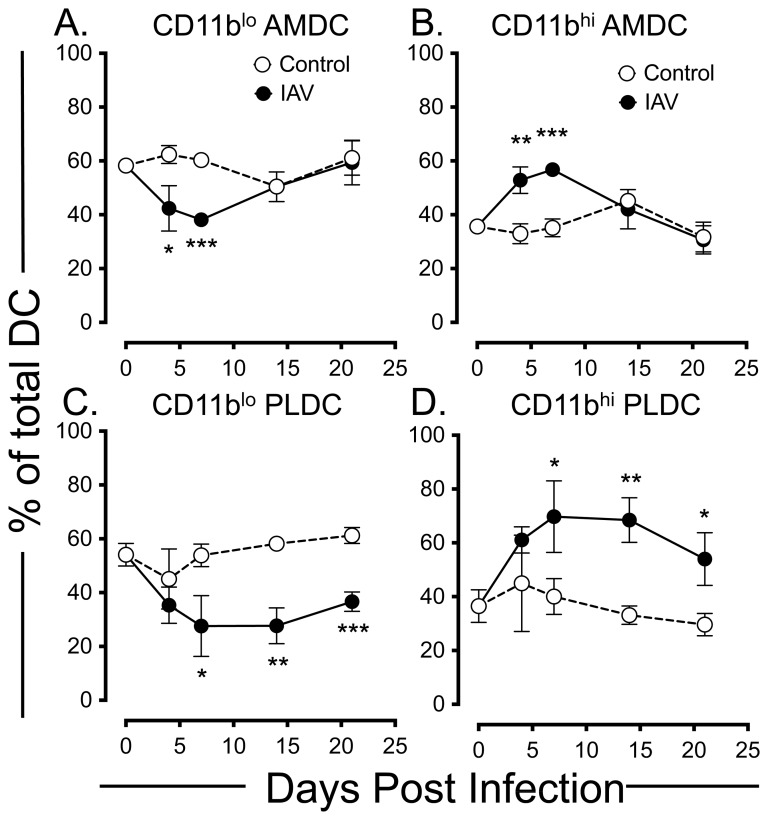
Time course of changes in the expression of CD11b on respiratory DC subsets in anatomical compartments of the respiratory tract following IAV infection. *(A and B)* Percentage frequency of CD11b^lo^ AMDC *(A)* and CD11b^hi^ AMDC *(B)* amongst total AMDC (gated as per Fig. 4A) in IAV infected (closed circles) and control mice (open circles). *(C and D)* Percentage frequency of CD11b^lo^ PLDC *(A)* and CD11b^hi^ PLDC *(B)* amongst total PLDC (gated as per Fig. 4D) in IAV infected (closed circles) and control mice (open circles). Data are means +/− SEM for 3 independent infection experiments using pools of tissue from 3 to 4 mice for each experiment. *  =  *p*<0.05; **  =  *p*<0.01; ***  =  *p*<0.001.

In addition to anatomical location, we and others have shown that the myeloid marker CD11b functionally divides mouse lung DC, with CD11b^hi^ and CD11b^lo^ DC subsets showing different rates of capture and trafficking of inhaled antigens in the steady-state and during allergic inflammatory airways disease [15], [16], [30]. Furthermore, the CD11b^lo^ DC subset has been shown to be important for clearance of IAV and to have distinct functional properties in terms of T cell recruitment and activation [22], [23]. In the current study, analysis of CD11b expression on respiratory tract DC populations following IAV infection showed a compartmentalised change. In the airway mucosa, IAV infection induced a significant decrease in the percentage of CD11b^lo^ AMDC (Fig. 5A), and corresponding increase in the percentage of CD11b^hi^ AMDC (Fig. 5B) at d4 and d7 p.i., returning to control levels for each subset at d14 p.i. Similarly, IAV infection also induced a decrease in the percentage of CD11b^lo^ PLDC (Fig. 5C), and increase in the percentage of CD11b^hi^ PLDC at d7 p.i. (Fig. 5D). However, in contrast to AMDC, changes in CD11b expression on PLDC persisted, remaining significantly changed from control PLDC until d21 p.i. (Fig. 5C and 5D).

In summary, IAV infection induced a transient derangement of AMDC percentages and CD11b expression that generally resolved by d14 p.i., whereas these alterations persisted in PLDC. Therefore, restoration of homeostasis of DC populations following IAV infection is rapidly regulated at the mucosal surfaces of the conducting airways, but remains disrupted in the lung parenchymal compartment.

### Expression of co-stimulatory markers on respiratory tract DC following acute IAV infection

**Figure 6:**
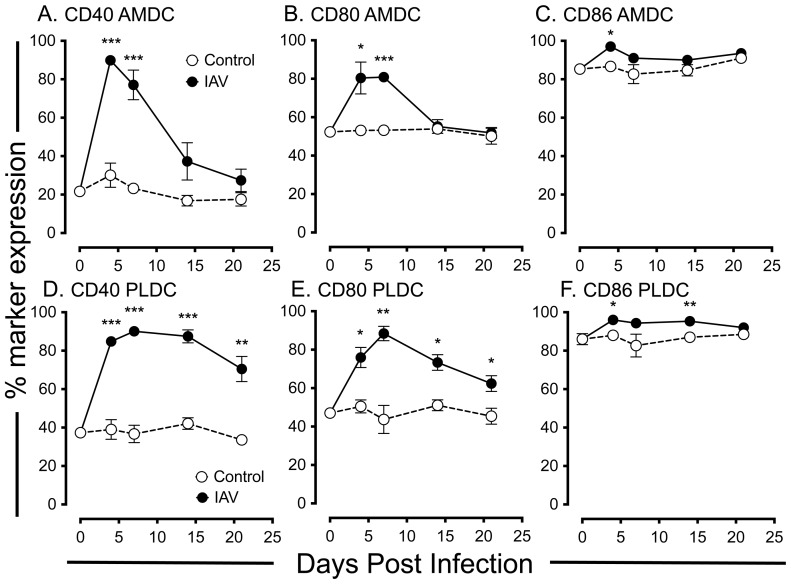
Expression of the co-stimulatory molecules CD40, CD80 and CD86 on respiratory DC subsets in anatomical compartments of the respiratory tract following IAV infection. *(A–C)* Time-course of expression of CD40 *(A),* CD80 *(B)* and CD86 *(C)* on AMDC expressed as a percentage of cells expressing each marker of the total AMDC population (gated as per Fig. 4A). *(D–F)* Time-course of expression of CD40 *(D),* CD80 *(E)* and CD86 *(F)* on PLDC expressed as a percentage of cells expressing each marker of the total PLDC population (gated as per Fig. 4D). Data are means +/− SEM for 3 independent infection experiments using pools of tissue from 3 to 4 mice for each experiment. The percentage expression of each marker was calculated based on histogram gates set using matching isotype control antibodies for each marker (Fig. S2) *  =  *p*<0.05; **  =  *p*<0.01; ***  =  *p*<0.001.

We next examined the expression of the co-stimulatory markers CD40, CD80 and CD86 on AMDC and PLDC following IAV infection as indicators of cellular activation status. We have previously shown that CD40 is an early activation marker of AMDC, being upregulated in the early stages of allergic airways disease [16]. For AMDC, the percentages of cells expressing CD40 (Fig. 6A) and CD80 (Fig. 6B) were significantly increased at d4 p.i. when compared to control AMDC (Fig. S2), generally returning to control levels by d14 p.i. Although baseline expression levels of CD86 were constitutively high on AMDC, expression of this marker was also significantly upregulated at d4 p.i., returning to control levels by d7- d14 p.i. (Fig. 6C). For PLDC, CD40 (Fig. 6D) and CD80 (Fig. 6E) were upregulated at d4 p.i. (Fig. S2) and, in contrast to AMDC, remained elevated until d21 p.i. Again, as for AMDC, CD86 expression was constitutively high on PLDC and was further upregulated from d4 to d14 p.i., returning to normal levels by d21 p.i. (Fig. 6F). Expression of these co-stimulatory markers was equally distributed amongst the CD11b^lo^ and CD11b^hi^ AMDC and PLDC subsets (data not shown).

In summary, AMDC showed a rapid and transient increase in co-stimulatory molecules following IAV infection that returned to baseline levels by d14 p.i., whereas PLDC showed a persistent increase in these molecules on both the CD11b^lo^ and CD11b^hi^ subsets. Therefore, control of DC activation following IAV infection is rapidly regulated in the airway mucosa, but remains dysregulated in the lung parenchymal compartment.

### Frequency and activation status of respiratory tract APC populations following early-life IAV infection

**Figure 7:**
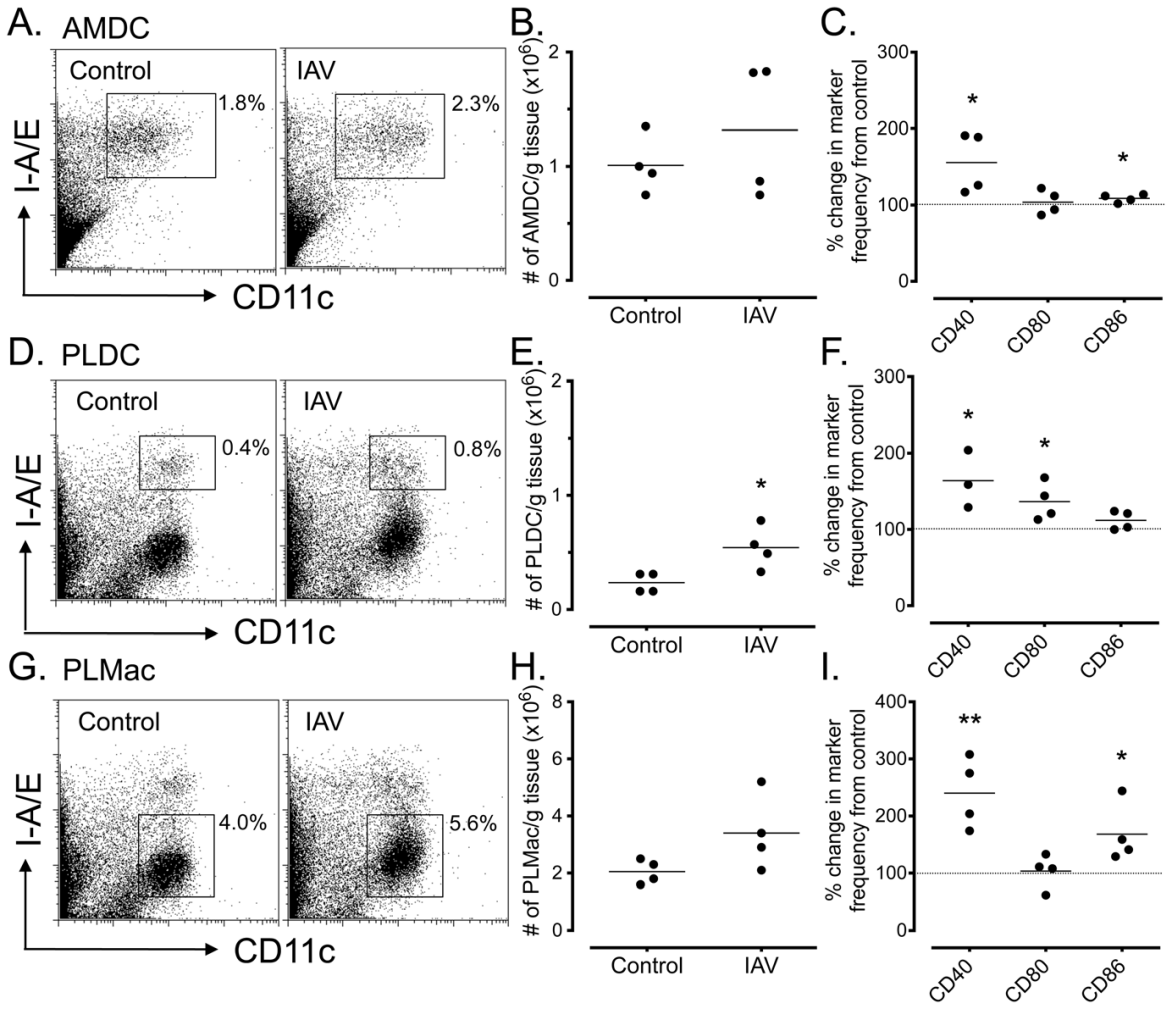
Long-term changes in total cell numbers of, and co-stimulatory molecule expression by, of respiratory APC populations following IAV infection in early life. Juvenile (28 day old) BALB/c mice were infected i.n. with a weight-adjusted dose of IAV as described in Materials and Methods, then respiratory tissues harvested 35 days later as 8 week-old adults. *(A)* Representative FACS profiles showing gating for AMDC in tracheal tissue of control (left) and IAV infected mice (right). *(B and C)* Total AMDC numbers *(B)* and percentage changes in co-stimulatory marker expression *(C)*, expressed a percentage change from control mice. *(D)* Representative FACS profiles showing gating for PLDC in parenchymal lung tissue of control (left) and IAV infected mice (right). *(E and F)* Total PLDC numbers *(E)* and percentage changes in co-stimulatory marker expression *(F)*, expressed a percentage change from control mice. *(G)* Representative FACS profiles showing gating for PLMac in parenchymal lung tissue of control (left) and IAV infected mice (right). *(H and I)* Total PLMac numbers *(H)* and percentage changes in co-stimulatory marker expression *(I)*, expressed a percentage change from control mice. Data are shown for 4 independent infection experiments, using pools of tissue from 4 to 5 mice for each experiment. *  =  *p*<0.05; **  =  *p*<0.01.

We next addressed the question of the long-term impact of IAV on respiratory tract APC populations after early-life virus infection. To investigate this, post-weaning (28 day-old) BALB/c mice were infected with a weight-adjusted dose of IAV as described in the Materials and Methods, and tracheal and lung tissue isolated 35 days later (i.e. as 8 week-old adults) to examine AMDC and PLDC cell frequencies and their activation status. Early-life IAV infection resulted in a clinical syndrome that was very similar to that shown for adult mice (see Fig. 1B), with clinical scores peaking and declining at similar time-points p.i. (data not shown). Analysis of tracheal tissue of mice infected at 28 days of age and analysed as adults showed no significant difference in percentages or total numbers of AMDC compared to control mice (Fig. 7A and 7B), however significant increases were observed in the percentage of cells expressing CD40 (*p*<0.01) and CD86 (*p*<0.01) (Fig. 7C). In contrast, analysis of peripheral lung tissue showed significantly elevated percentages (*p*<0.01, data not shown) and total numbers (*p*<0.05) of PLDC compared to control mice (Fig. 7D and 7E), and significantly up-regulated percentages of cells expressing CD40 and CD80 (*p*<0.01) (Fig. 7F). Analysis of PLMac showed a significant increase in the percentage of cells (*p*<0.05, data not shown), but no significant increases in the percentages or total numbers of cells (Fig. 7G and 7H), and significant increases in the proportions of cells expressing CD40 and CD80 (Fig. 7I). Furthermore, a small but significant decrease in the intensity of MHC Class II expression was observed on PLDC (p<0.01), and a significant increase on PLMac (p<0.001) (Fig. S3).

In summary, early life IAV infection induced increases in PLDC numbers and increased expression of co-stimulatory molecules on AMDC, PLDC and PLMac that persisted into adulthood. These data indicate that restoration of respiratory APC homeostasis is persistently disrupted if IAV infection occurs in early life.

## Discussion

This study examined the acute and long-term effects of A/PR8/34 H1N1 IAV infection on the depletion, reconstitution kinetics and activation state of DC populations in mucosal and parenchymal lung compartments. Uniquely, these were examined both in adult and juvenile mice for airway mucosal and parenchymal lung tissue sites, representing the two major anatomical compartments of the respiratory tract. We have argued previously that these sites differ in their induction and effector immunoregulatory functions in the mouse RT [15], [16]. In this study, we have explored the hypothesis that these sites will respond differentially to IAV infection, and that the impact of IAV on immunological homeostasis in the RT will vary as a function of the age of infection.

An adult BALB/c mouse model of IAV infection was established utilising a sub-lethal dose of A/PR8/34, in which clinical signs peaking at days 8 to 9 p.i., with viral titres and lung histopathology reverting to background levels by day 14 p.i. Influenza infection resulted in significant and physiologically important deficits in lung mechanics of the conducting airways, peripheral airways and lung parenchyma that were maintained at high lung volume. Mice showed altered lung physiological responses at the peak of IAV infection, which was associated with the maximum of the neutrophil influx into alveolar space. These data are consistent with previous studies using the A/Mem/1/71(H3N1) strain of influenza virus in BALB/c mice, which showed heightened AHR at day 4 p.i. which had normalised by day 20 p.i. [31]. The acute phase of viral infection was also characterised by peak influxes of lymphocytes and neutrophils into BAL fluid as well as IFNα, the neutrophil chemoattractant KC (CXCL1) and IL-12 p70, which declined by day 14 p.i. The neutrophil response in particular was of interest, as these cells had not return to baseline levels by day 14 when virus had been cleared, and began to increase again by day 14. Interestingly, this second wave of neutrophil influx occurred without a concurrent increase in KC or detectable viral titres in lung tissue, suggesting a secondary recruitment response independent of these factors was taking place. The reason for this is unclear, as we saw no indirect evidence of secondary bacterial infection in the mice, further supported by a lack of Type 1 IFN responses in BAL fluids after day 9 p.i., however this would need to be confirmed by bacteriology. However, the bi-phasic neutrophil response did correlate with a bi-phasic G-CSF response in BALF. This growth factor is a potent regulator of haemopoesis, and mediates neutrophil activation and survival [32]. It is possible that failure to correctly regulate G-CSF in the resolution phase of disease may lead to persistent neutrophil recruitment and activation and incomplete resolution of the inflammatory response. Recently, Narasaraju *et al.* showed a role for macrophages in limiting neutrophil influxes into lavage fluids following PR8 infection [33]. These late and persistent increases in inflammatory cells in BAL suggest that inflammation may never be fully resolved after IAV infection in BALB/c mice, which is consistent with what has been previously proposed and which may in part explain post-infective bronchial hyperreactivity that may persist in humans for weeks or months after influenza infection [34], [35].

A key objective of the current study was to examine the kinetics of changes in numbers and activation status of DC, in different anatomical compartments of the mouse respiratory tract after influenza infection. This was based on our previous mouse studies showing that AMDC displayed more rapid turnover rates and were activated early in response to aeroallergen challenge compared to their PLDC counterparts [15], [16]. We reasoned that the responses of DC in these two compartments would differ given that AMDC are in intimate contact with airway epithelial cells, which represent the first site of influenza infection and replication, whereas PLDC reside within the alveolar septal walls that represent a distinct microenvironment that may be influenced by inflammatory responses later in the course of infection [36]. Dendritic cells play an important role in the initiation of anti-viral T cell responses, rapidly migrating to draining lymph nodes during early infection and being critical for the activation of both CD4^+^ and CD8^+^ viral-specific T cell responses that are essential for viral clearance and resolution of inflammation [27], [36], [37]. The findings of this study are in accordance with this, as we found that AMDC were being activated to express costimulatory markers early after infection, that correlated with induction of high levels of IAV-specific CD8^+^ T cell proliferation and IFNγ production in draining lymph nodes at day 4 p.i. (Wikstrom, Stumbles, unpublished observations). Interestingly, associated with this was a significant decrease in the numbers of AMDC at day 4 p.i. but recovery by day 14 p.i., suggesting either cell death, or enhanced migration of AMDC to draining lymph nodes after IAV infection, consistent with the previous findings of others for pulmonary viral infections [37]. Previously we have shown that mouse AMDC and PLDC can be subdivided based on expression of the myeloid marker CD11b into CD11b^hi^ and CD11b^lo^ subsets that show different rates of capture of inhaled antigens *in vivo* [16], [30]. Others have shown that the CD11b^lo^ subset, that co-expresses the surface marker CD103, is important in capturing viral antigens and migrating to draining lymph nodes for the activation of CD4^+^ and CD8^+^ T cells. This capacity also resides within the CD11b^hi^ subset, although to a lesser extent [26]. Furthermore, CD11b^lo^ and CD11b^hi^ DC in the mouse RT have been shown to express differing arrays of chemokines and have been proposed to play differing roles in lung homeostasis, with the CD11b^lo^ (CD103^+^) subset expressing tight junction proteins and may play a major regulatory role in allergen-induced lung inflammation [22], [23]. The findings in this study that the relative proportions of CD11b^hi^ PLDC remain persistently elevated amongst the PLDC population after viral clearance. The reason for this disrupted balance in CD11b-expressing subsets was not determined in the current study, but may relate to altered recruitment kinetics or local maturation of CD11b^hi^ and CD11b^lo^ subsets. In this regard, Lin et al., described a population of inflammatory CCR2^+^ monocyte-derived DC recruited during acute IAV infection, that have a similar phenotype to the PLDC observed in our study (CD11c+ MHC Class II^+^ CD11b^hi^) [38]. Thus, these cells may be contributing to the enhanced proportions of CD11b^+^ PLDC that persist following IAV infection in our study. Disruptions to the balance of CD11b-expressing PLDC subsets, along with persistent elevation of co-stimulatory molecule expression on these cells, may have important implications for immune homeostasis at this site given that maintaining a balance in these subsets of DC is likely to be important for the correct regulation of T cell immunity to inhaled allergens. In this regard, it is of interest to note that IAV infection has been associated with enhanced IgE production and CD4^+^ T cell sensitisation to inhaled allergens, an effect linked to altered function of respiratory dendritic cells [7], [39]–[41]. Furthermore, IAV has been shown to disrupt the induction of T and B cell tolerance to inhaled antigens that normally occurs following exposure to inert proteins in immunologically naïve mice [8]. It is interesting to speculate that these observations may be linked to the findings in the current study of persistently increased proportions of activated CD11b^hi^ PLDC following IAV infection, that may act to promote aberrant local CD4^+^ T cell activation to inhaled allergens in lung tissue.

Finally, we examined the impact of IAV infection in juvenile mice at the age of 28 days old, representing the post-weaning age of human infants. Juvenile mice infected with a weight-adjusted, adult-equivalent dosage of IAV developed a clinical disease of similar severity to adults, with peak clinical signs and resolution of symptoms occurring at the same time points. When these mice were examined at day 35 p.i., an age at which is mice are considered to have reached adult maturity, we observed persistent and significant increases in the numbers and activation status of parenchymal lung DC and Mac populations, but not in their airway mucosal counterparts. This was particularly evident for the PLMac populations, which displayed marked increases in MHC Class II, CD40 and CD86 expression above control mice, and for PLDC that displayed increased numbers and persistent CD40 and CD80 upregulation. Activation of PLMac was also observed in the adult model prior during the course of IAV infection, most likely as a result of TLR7 binding by viral components and NLR (NOD-like receptor) inflammasome activation [42]. Whether persistent PLMac and PLDC activation in juvenile and adult mice is a result of sustained TLR or NLR signalling, or some other sustained inflammatory response is unclear. However, this could have important down-stream consequences for immune homeostasis at in the lung, given that interactions between macrophages and DC are important for dampening DC function and T cell reactivity in parenchymal lung tissue and that long-lived APC expressing elevated levels of MHC Class II could act as depots for allergen persistence and reactivation of allergen-specific T cells [43], [44].

In conclusion, in this study we have demonstrated that A/PR8/34 H1N1 IAV infection of BALB/c mice has differential effects on DC populations in differing anatomical locations of the respiratory tract, with persistent derangement in the numbers and activation states of DC in the parenchymal lung compartment as compared to the airway mucosa. Furthermore, these disruptions persisted for several weeks after clearance of the virus in adult mice, and persisted into adulthood in mice that were infected with IAV in early life. These data indicate that IAV has a severe and long-term impact on the balance and activation state of respiratory DC and other APC subsets, potentially disrupting the fine balance of immunological homeostatic mechanisms required for the prevention of respiratory inflammatory diseases to inert antigens.

## Materials and Methods

### Mice and Viral Inoculations

#### Ethics statement

All animal experiments were conducted in strict accordance with the recommendations of the National Health and Medical Research Council of Australia, Guidelines to Promote the Wellbeing of Animals used for Scientific Research. All procedures were approved by the Telethon Institute for Child Health Research Animal Experimentation Ethics Committee (permit numbers: 139 and 256) and Murdoch University Animal Ethics Committee (permit number: N2569/13). Intranasal (i.n.) viral inoculations were performed under light inhaled Isofluorane anaesthesia. Mice were sacrificed by i.p. injection of 100µl of Phenobarbitone Sodium performed under inhaled Isofluorane anaesthesia, with all efforts made to minimise animal suffering.

Specific pathogen free female BALB/c mice were obtained from the Animal Resources Centre (Perth, W.A., Australia) and used at either 28 days (juvenile) or 8 weeks (adult) of age. The mouse-adapted influenza H1N1 A/PR/8/34 virus was from the American Type Tissue Culture Collection and prepared in allantoic fluid of 9-day old embryonated hens eggs. Stock virus was sub-passaged through Mardin-Darby canine kidney (MDCK) cells in Dulbecco's modified Eagle's medium (DMEM; Gibco, Sydney, Australia), harvested as tissue culture supernatant and viral titres determined by cytopathic effects on MDCK cells and expressed as the mean log_10_ tissue culture infective dose that kills 50% of the cells (TCID_50_) over a 5-day incubation period. Adult mice were inoculated intranasally (i.n.) under light inhalation anaesthesia with 0.5×10^2^ TCID_50_ PR8 diluted in 50µl DMEM [45]. Juvenile mice were obtained post-weaning and inoculated i.n. using a weight-for-age adjusted volume of 0.5×10^2^ TCID_50_ PR8/50µl adjusted to 2.5µl per gram body weight. Mock-infected control mice received matched volumes of virus-free DMEM tissue culture medium by i.n. inoculation.

### Animal Monitoring and Clinical Assessments

Mice were weighed daily during the acute period of infection (d0 to d14) and then every second day until day 21. Clinical disease scores were also assessed according ot the following criteria: 0-healthy; 1-barely ruffled fur; 2-ruffled fur, active; 3-ruffled fur, inactive; 4-ruffled fur, inactive, hunched. Mice were euthanized at indicated time points and lung tissue harvested for assessment of viral titres. Lung-tissue viral titres were determined by the TCID_50_ assay described above, using 20% dilutions of clarified lung tissue homogenates. Results are expressed as log_10_ TCID_50_/100µl of lung tissue homogenate. Broncho-alveolar lavage fluid (BALF) was harvested by slowly infusing and withdrawing 1 ml of PBS containing 20mg/ml bovine serum albumin (CSL, Victoria, Australia) and 35 mg/ml lidocaine (Sigma, St Louis, USA) from the lungs three times, and the cells pelleted and prepared for total cell counts and differential cell counts as previously described [46]. Briefly, the percentage of each cell type as identified by Leishman's stain (macrophage, neutrophil, lymphocyte) was calculated as a proportion of 300 counted cells, and this figure used to derive total numbers of each subset based on the total BALF cell count. The BALF supernatants were stored at -80°C for cytokine analysis. All cytokines were measured in undiluted BALF using a Bio-Plex Pro Mouse Cytokine Grp 1 Panel 23-Plex assay and Bio-Plex MAGPIX plate reader (BIO-RAD, USA) apart from acid stable Type I IFN (IFNα), which was measured by bioassay using encephalomyocarditis virus-induced cytopathic effect (CPE) of L929 monolayers as previously described [47].

### Lung Function Testing

Lung mechanics were measured as described previously [48]. Briefly, mice (4 per experimental group) were anaesthetised by i.p. injection of a solution containing 40 mg.mL^−1^ ketamine and 2 mg.mL^−1^ xylazine at a dose of 0.1 mL.10 g^−1^ body weight. Mice were surgically tracheotomised and connected to a mechanical ventilator (tidal volume, 8 mL.kg-1; frequency, 450 breaths.min-1, PEEP, 2 cmH_2_O). Following standardisation of lung volume history the ventilator was paused and a pseudorandom oscillatory signal (4-38 Hz) was delivered to the tracheal cannula by a loudspeaker via a wavetube of known impedance. A positive pressure was then applied via the wavetube in order to slowly (15–20s) inflate the lung up to 20 cmH_2_O transrespiratory pressure. The oscillatory signal was applied throughout this manoeuvre in order to track changes in lung mechanics from functional residual capacity (FRC) to total lung capacity (TLC). A respiratory system impedance spectrum was then generated for each 0.5 s data epoch of the inflation manoeuvre. Data from each of these spectra was then fit to a 4 parameter mathematical model with constant phase tissue impedance which allowed us to partition lung mechanics into parameters representing airway resistance (Raw, resistance of the conducting airways), tissue damping (G, resistance of the small peripheral airways were airflow occurs by diffusion) and tissue elastance (H, stiffness of the lung parenchyma) [49].

### Isolation and Preparation of Respiratory Tract Tissues

Lungs were perfused to isolate tracheal (airway mucosal) and parenchymal lung tissue and prepare single cell suspensions as previously described [50]. In brief, trachea or lungs were collected from pools of five mice and single-cell suspensions prepared by type IV collagenase digestion (1.5 mg/ml; Worthington Biochemical, Lakewood, NJ) with type I DNAse (0.1 mg/ml; Sigma Aldrich). DLN (upper paratracheal and parathymic) were pooled separately from the same groups of mice, finely chopped with a scalpel, and digested with type IV collagenase and type I DNAse. All digestions and washes were performed in glucose sodium potassium buffer (11 mM D-glucose, 5.5 mM KCl, 137 mM NaCl, 25 mM Na_2_HPO_4_, 5.5 mM NaH_2_PO_4_.2H_2_O) with debris and RBCs removed as previously described [51].

### Analysis of Cell Surface Markers by Flow Cytometry

After preparation of single-cell suspensions, FcR were routinely blocked using 2.4G2 (BD Biosciences) for 10 min on ice to prevent non-specific binding of phenotyping antibodies subsequently added. Airway, lung and draining lymph node DC populations were identified in tracheal digests using combinations of fluorochrome labelled mAbs (all from BD Biosciences, NSW, Australia except where indicated) to mouse CD11c (clone N418), I-A/I-E (clone 2G9), CD205 (DEC205; Serotec, Oxford, UK), CD11b (clone M1/70), CD40 (clone 3/23), CD80 (clone 16-10A1) and CD86 (clone GL1). All labelling was performed in glucose sodium potassium buffer containing 0.2% BSA for 30 min on ice. All Abs were used as direct conjugates to FITC, Phycoerythrin (PE), PE-Cy7, allophycocyanin (APC), APC-Cy7, or biotin as required. Where appropriate, biotinylated antibodies were detected with Streptavidin conjugated PE-Cy5 (BD Biosciences). Appropriately matched and conjugated IgG isotype controls (BD Biosciences) were used in all experiments, and cytometer compensation settings were adjusted using single-stained controls for each experiment. Samples were collected using a FACSCalibur or LSRII flow cytometer (BD Biosciences) and analyzed using FlowJo software (TreeStar, Ca, USA).

### Determination of Total Cell Counts

Total counts for AMDC, PLDC and PLMac were determined on the basis of tissue weight and total cell yield for each tissue (trachea and peripheral lung), and then calculated on the basis of the total percentage of each DC type as determined by FACS. Values are expresses as number of cells/g tissue according to the formula: (% frequency x total cells/g tissue)/100.

### Statistical Analysis

Two-tailed, unpaired Student's t tests assuming equal variance were employed to calculate significances (GraphPad Prism, CA, USA), with *p*-values <0.05 considered statistically significant. Statistical significance is indicated as follows: *  =  *p*<0.05; **  =  *p*<0.01; ***  =  *p*<0.001; ****  =  *p*<0.0001.

## Supporting Information

Figure S1 **CD205 and CD11c staining of respiratory tissues.** Tracheal (A) and parenchymal lung tissue (B and C) cells were prepared as per Methods, labelled for CD11c and CD205 and analysed by flow cytometry at the indicated time points after IAV infection. Representative dot plots of 3 experiments for each time point are shown, with AMDC (A), PLDC (B) and PLMac (C) gated as indicated.
(TIF)

Figure S2 **Expression levels of co-stimulatory markers on AMDC and PLDC at d4 post-IAV infection.** Adult BALB/c mice were infected with IAV and 4 days later (representing a time point of peak expression of each marker) tracheal and lung tissue were prepared for flow cytometry analysis for each marker on AMDC (A) and PLDC (B). Representative histograms (of 3 experiments) are shown for control and IAV infected mice, showing isotype controls (grey shaded) and specific marker (solid black) expression.
(TIF)

Figure S3 **Changes in MHC Class II (I-A/E) expression on respiratory APC of adult mice following IAV infection at 4 weeks of age.** Mice were infected with IAV at 4 weeks of age and respiratory tissue harvested 35 days later. The mean fluorescence intensity (MFI) of I-A/E expression on AMDC (A), PLDC (B) and PLMac (C) was then determined for IAV infected mice (black line) and control mice (grey shade), and expressed as a percentage change in MFI of IAV infected mice as compared to control mice (D). Histograms are representative of 4 independent experiments using pooled tissue from 5 mice in each, gated on DC populations as shown in Fig. S1. Panel D shows cumulative data of 4 independent experiments using pools of tissue from 5 mice in each experiment.
(TIF)
