# Supplementary figures and images for: Persistent and Compartmentalised Disruption of Dendritic Cell Subpopulations in the Lung following Influenza A Virus Infection
Source: PLoS One. 2014 Nov 14;9(11):e111520. doi: 10.1371/journal.pone.0111520 (PMC4232261; doi:10.1371/journal.pone.0111520)

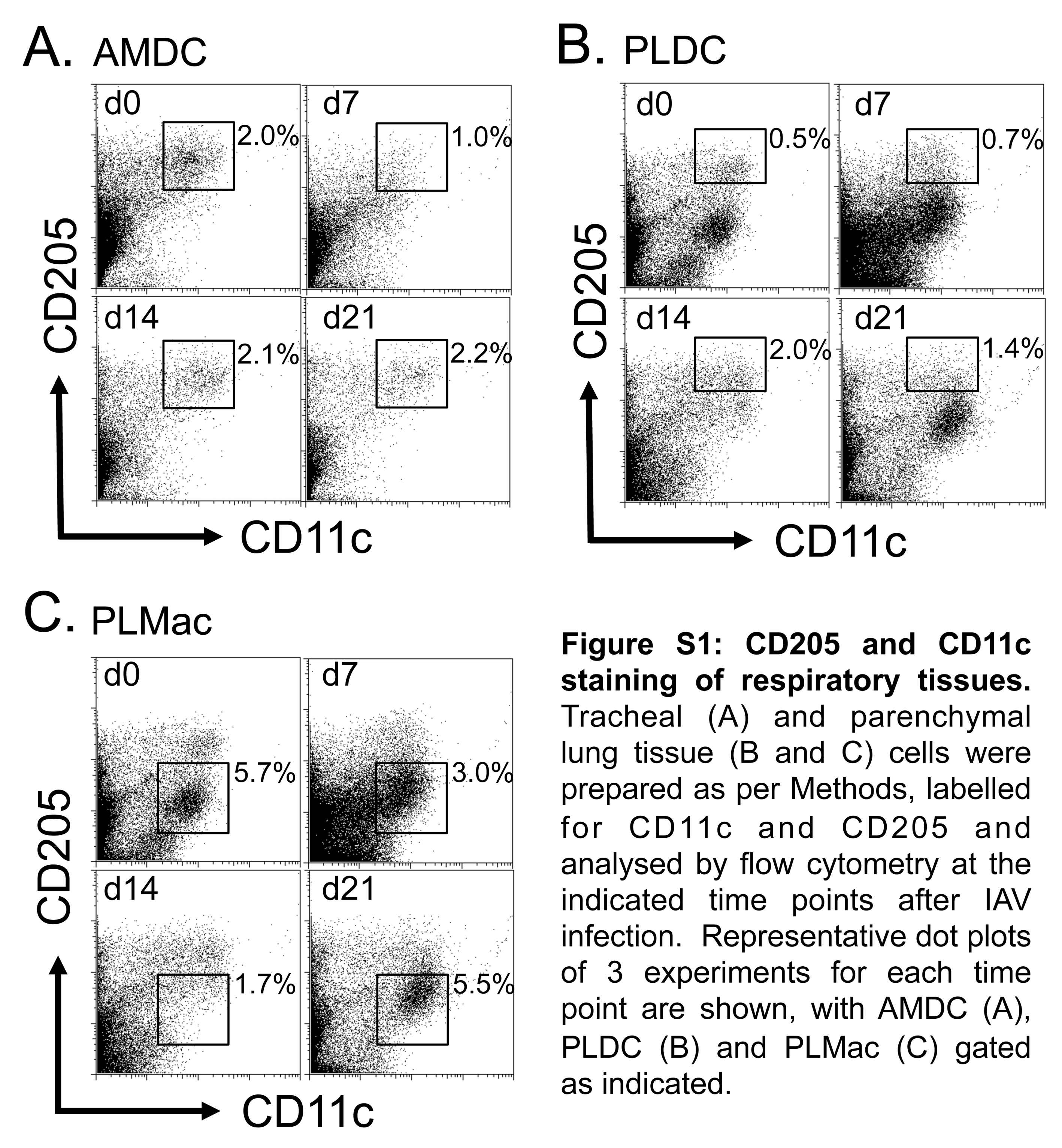

Supplement: Figure S1 — CD205 and CD11c staining of respiratory tissues. Tracheal (A) and parenchymal lung tissue (B and C) cells were prepared as per Methods, labelled for CD11c and CD205 and analysed by flow cytometry at the indicated time points after IAV infection. Representative dot plots of 3 experiments for each time point are shown, with AMDC (A), PLDC (B) and PLMac (C) gated as indicated. (TIF) [file pone.0111520.s001.tif]

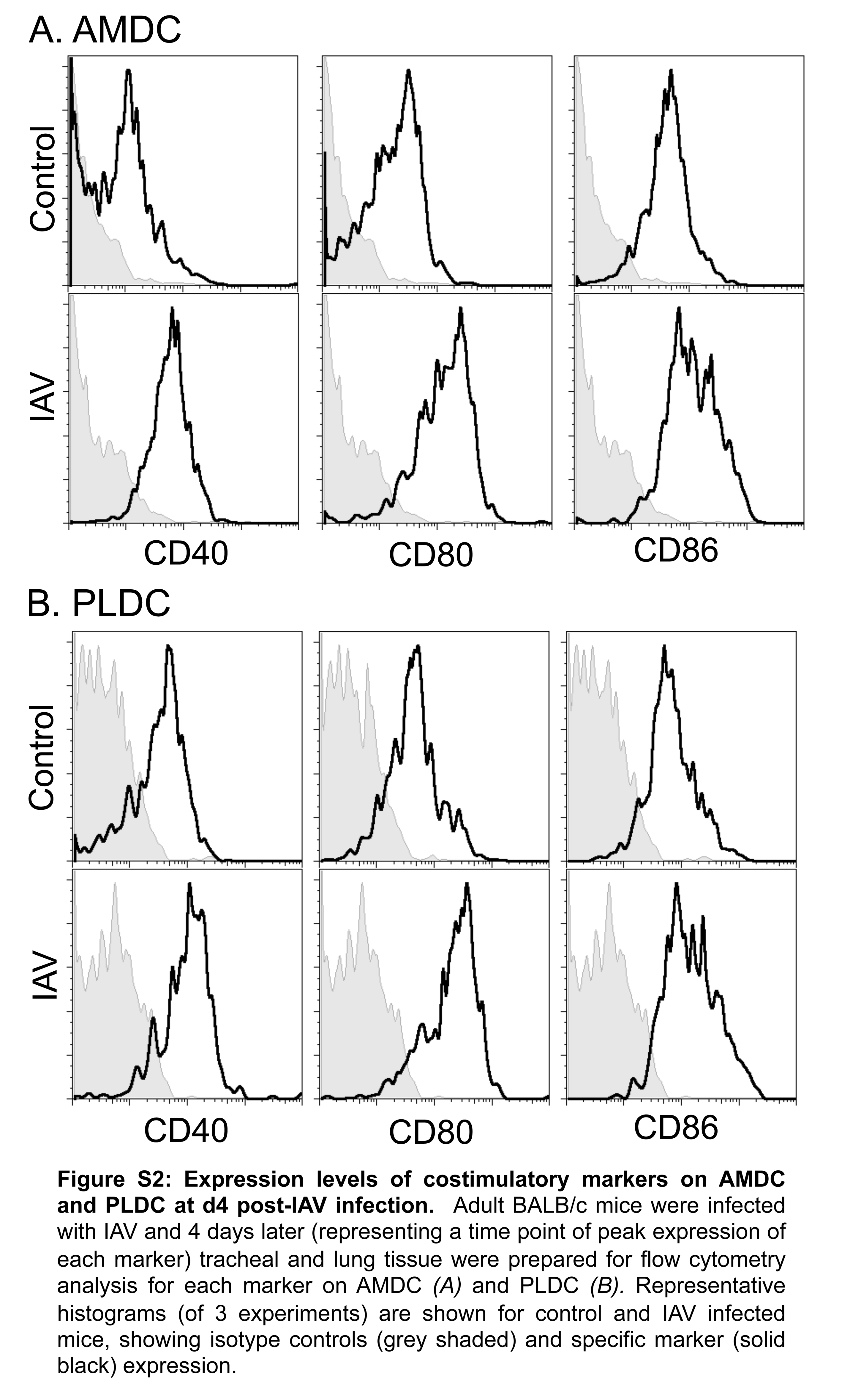

Supplement: Figure S2 — Expression levels of co-stimulatory markers on AMDC and PLDC at d4 post-IAV infection. Adult BALB/c mice were infected with IAV and 4 days later (representing a time point of peak expression of each marker) tracheal and lung tissue were prepared for flow cytometry analysis for each marker on AMDC (A) and PLDC (B). Representative histograms (of 3 experiments) are shown for control and IAV infected mice, showing isotype controls (grey shaded) and specific marker (solid black) expression. (TIF) [file pone.0111520.s002.tif]

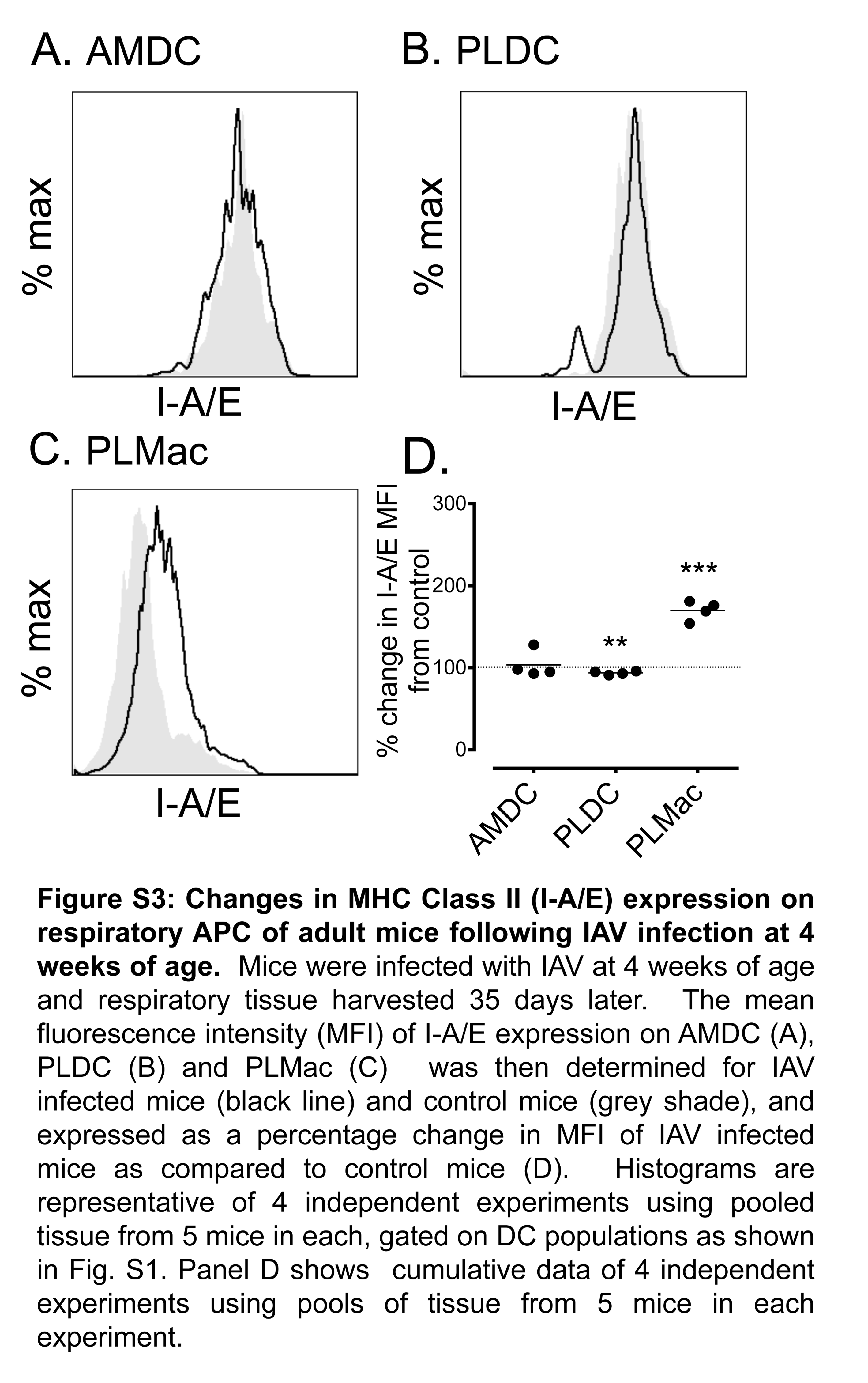

Supplement: Figure S3 — Changes in MHC Class II (I-A/E) expression on respiratory APC of adult mice following IAV infection at 4 weeks of age. Mice were infected with IAV at 4 weeks of age and respiratory tissue harvested 35 days later. The mean fluorescence intensity (MFI) of I-A/E expression on AMDC (A), PLDC (B) and PLMac (C) was then determined for IAV infected mice (black line) and control mice (grey shade), and expressed as a percentage change in MFI of IAV infected mice as compared to control mice (D). Histograms are representative of 4 independent experiments using pooled tissue from 5 mice in each, gated on DC populations as shown in Fig. S1. Panel D shows cumulative data of 4 independent experiments using pools of tissue from 5 mice in each experiment. (TIF) [file pone.0111520.s003.tif]
